# Association between controlling nutritional status score and mortality in older patients with dysphagia in Japan: **a** retrospective cohort study

**DOI:** 10.3389/fnut.2025.1682772

**Published:** 2025-11-18

**Authors:** Yan Wang, Caihong Sun, Xiuqing Tian, FangFang Fan

**Affiliations:** 1 Department of Intensive Care Unit, Rizhao People's Hospital, Rizhao, China; 2 Department of Cardiology, The First Affiliated Hospital of Shandong First Medical University & Shandong Provincial Qianfoshan Hospital, Jinan, China

**Keywords:** controlling nutritional status (CONUT) score, dysphagia, mortality, older patients, retrospective cohort study

## Abstract

**Background:**

Dysphagia confers elevated risks of adverse clinical outcomes. While the Controlling Nutritional Status (CONUT) score has emerged as a prognostic indicator of mortality in critically ill populations, its association with mortality risk among Japanese geriatric patients with dysphagia remains poorly characterized.

**Objective:**

This retrospective cohort study investigated the prognostic utility of the Controlling Nutritional Status (CONUT) score in geriatric Japanese patients with dysphagia.

**Methods:**

Clinical data from 236 consecutive dysphagia patients admitted to a tertiary care center (January 2014-January 2017) were analyzed. Nutritional risk stratification was performed using CONUT criteria. Mortality associations were assessed through multivariable Cox proportional hazards models, with subgroup analyses conducted to evaluate effect modification. Survival patterns were visualized using Kaplan-Meier methodology. A receiver operating characteristic curve analysis (ROC) was conducted to assess the predictive ability.

**Results:**

The cohort (median age 83 years, 59.7% female) demonstrated dose-dependent mortality relationships with CONUT severity. After full covariate adjustment, each unit CONUT increase corresponded to 15% elevated mortality risk (adjusted HR 1.15, 95% CI 1.08–1.23; *P* < 0.001). Graded associations were observed across nutritional risk strata vs. reference (CONUT 0–1): mild (2,3,4) HR 2.46 (1.02–5.91, *P* = 0.045), moderate (5,6,7,8) HR 2.91 (1.21–7.02, *P* = 0.017), and severe (9,10,11,12) HR 4.56 (1.84–11.3, *P* = 0.001). Median survival durations decreased progressively: 716 days (mild), 362 days (moderate), and 106 days (severe). Further ROC curve analysis demonstrated that CONUT (AUC 0.714, 95% CI 0.649–0.779) is an effective tool to predict mortality in older patients with dysphagia.

**Conclusion:**

CONUT score independently predicts all-cause mortality in Japanese elderly with dysphagia, demonstrating monotonic risk gradients across nutritional severity categories. The absence of significant interaction effects in subgroup analyses reinforces the robustness of this association.

## 1 Introduction

Dysphagia is characterized by difficulties in moving food from the mouth to the stomach (1, 2). Its incidence and prevalence rise with age due to declining physiological functions, particularly in individuals with neurological conditions like Parkinson's disease and cerebrovascular events (3). Among older adults, the prevalence of dysphagia ranges from 10% to 33%, exceeding 50% among hospitalized patients and nursing home residents (3–5). Dysphagia not only results in increased oral secretions but also leads to serious complications such as aspiration pneumonia, malnutrition, and dehydration, thereby elevating hospitalization rates and mortality risks in the elderly (6, 7). Consequently, timely identification and intervention for elderly individuals with dysphagia are imperative.

Nutritional status significantly impacts the prognosis of elderly individuals with dysphagia (8). Malnutrition can compromise immune function, impede wound healing, and increase the risk of infection, thereby affecting patient recovery (3). Precise evaluation of nutritional status and the formulation of personalized nutritional interventions plans are pivotal for to enhancing patient outcomes (9).

Commonly utilized nutritional assessment tools encompass anthropometric measurements, biochemical markers, dietary evaluations, and comprehensive nutritional assessment instruments (8, 10). Among these, the CONUT (Controlling Nutritional Status) score emerges as a straightforward, rapid, and cost-effective approach for evaluating nutritional status by integrating serum albumin levels, total cholesterol concentrations, and lymphocyte counts (11). Widely adopted in oncology and cardiology for assessing nutritional risk and forecasting clinical outcomes (12–14), the CONUT score has increasingly attracted attention in geriatrics. Research indicates a strong correlation between the CONUT score and frailty, inflammatory status, and body composition in elderly patients (11). Furthermore, the CONUT score serves as a predictive tool for mortality risk, duration of hospitalization, and postoperative complications among elderly patients (15, 16).

However, The utility of the CONUT score in elderly patients with dysphagia is not well-investigated. This study seeks to assess the applicability of the CONUT score in evaluating the nutritional status of this demographic. Through examining the correlation between the CONUT score and clinical, nutritional, and prognostic parameters in elderly patients with dysphagia, our objective is to offer clinicians a precise and efficient tool for nutritional assessment to identify high-risk individuals and guide clinical monitoring.

## 2 Materials and methods

### 2.1 Data source

The research data provided by Masaki Shigenori et al. were sourced from the Dryad Digital Repository (17), a platform that facilitates open access to primary data. Following the guidelines outlined in the Dryad Terms of Service, we analyzed the data package named “Baseline C-reactive protein, albumin level, and life prognosis in Dryad,” which can be accessed at https://doi.org/10.5061/dryad.gg407h1. Ethical approval for this retrospective study utilizing de-identified data was obtained from the Institutional Review Board of Miyanomori Memorial Hospital. The IRB granted a waiver of informed consent requirements based on the study's retrospective design and the analysis of anonymized data extracted from the database. All data handling and analytical procedures complied with relevant ethical standards, including the Declaration of Helsinki. In our analysis, individuals with incomplete CONUT scores (*n* = 18) were excluded, resulting in a final study cohort of 236 participants.

### 2.2 Study design and participants

This retrospective cohort study investigated clinical outcomes in geriatric patients with confirmed severe dysphagia receiving percutaneous endoscopic gastrostomy (PEG) or total parenteral nutrition (TPN) at a tertiary care center from January 2014 to January 2017. Clinical evaluation of dysphagia severity was conducted through standardized multidisciplinary assessment (physicians, nursing specialists, speech-language pathologists) complemented by videofluoroscopic swallowing studies (VFSS), with functional assessment scale scores confirming profound swallowing impairment in all enrolled subjects. Exclusion criteria encompassed: (1) terminal malignancy with life expectancy < 6 months, (2) PEG placement for non-nutritional indications (e.g., gastric decompression), and (3) prior gastrostomy procedures preceding the study period. These exclusions help to avoid the confusion of imminent mortality, non-nutritive uses, and previous treatment history on the nutritional status and outcome. The institutional review board of Miyanomori Memal Hospital granted ethical approval and waived informed consent requirements due to retrospective anonymised data analysis, with all interventions complying with current clinical guidelines.

### 2.3 Procedures

The selection between PEG feeding and TPN was determined through multidisciplinary consultations involving attending physicians and patients (or their legal representatives). Nutritional interventions were tailored according to individualized clinical assessments documented in treatment protocols. Demographic and clinical parameters extracted from electronic medical records included: chronological age (18), biological sex, comorbidities [cerebrovascular disorders (18), advanced dementia (19), aspiration pneumonia (20), ischemic cardiomyopathy], utilization of non-tunneled central venous catheters (NT.CVC), PEG implantation status (17, 21), functional oral intake recovery, and hematological biomarkers. Laboratory parameters were performed within the 7-day pre-intervention window preceding nutritional support initiation. The primary objective focused on quantifying procedure-associated mortality during the predefined observation window. CONUT scores were calculated using three hematological parameters: serum albumin concentration; absolute peripheral blood lymphocyte count; and total cholesterol levels. Nutritional risk stratification was implemented based on established cutoff values: absence of malnutrition (score 0–1); mild (2–4); moderate (5–8); and severe malnutrition (9–12); detailed information was provided in Table 1.

**Table 1:** Definition of controlling nutritional status (CONUT) score.

**Parameter**	**Malnutritional state**			
	**None**	**Mild**	**Moderate**	**Severe**
ALB (g/L)	≥ 35.0	30.0–34.9	25.0–29.9	< 25.0
Score	0	2	4	6
TLC ( × 10^9/*L*^)	≥ 1.60	1.20–1.59	0.80–1.19	< 0.80
Score	0	1	2	3
TC (mg/dl)	≥ 180	140–179	100–139	< 100
Score	0	1	2	3
Total CONUT Score	0–1	2–4	5–8	9–12

ALB, serum albumin; TL, total lymphocyte count; TC, total cholesterol level; Total CONUT score = ALB score + TL score + TC score.

### 2.4 Statistical analysis

Secondary analyses leveraged open-access registry data from Dryard. Continuous variables were presented as mean ± standard deviation (SD) for normally distributed data or median with interquartile range (IQR) otherwise. Categorical variables were expressed as absolute frequencies and proportions. Between-group comparisons were performed using ANOVA or the Kruskal-Wallis test for continuous variables, as appropriate, whereas categorical variables were analyzed via χ^2^ tests. To assess the independent association between CONUT scores and mortality risk, multivariate Cox proportional hazards models were constructed to estimate adjusted hazard ratios (HRs) with corresponding 95% confidence intervals (CIs). Restricted cubic spline (RCS) regression was implemented to examine potential association between CONUT scores and mortality outcomes. Survival probabilities across CONUT subgroups were visualized through Kaplan-Meier curves, and stratified analyses were conducted across clinically relevant subgroups including age, sex, ischemic heart disease (IHD), cerebrovascular disorders, severe dementia, aspiration pneumonia, percutaneous endoscopic gastrostomy (PEG) placement, nasoenteric tube/catheter (NT.CVC) use, oral intake recovery status, and energy intake levels. The predictive power of the CONUT score for mortality was assessed using the Receiver Operating Characteristic (ROC) curve, with identification of its optimal cut-off value for clinical risk stratification.All statistical procedures were executed using R software (version 4.2.2; R Foundation for Statistical Computing) and Free Statistics software (version 2.1.1), with a two-tailed α level of 0.05 defining statistical significance.

## 3 Results

### 3.1 Baseline characteristics of participants

This investigation analyzed 236 Japanese geriatric patients with dysphagia (95 males, 141 females), categorized into four CONUT-defined nutritional risk strata: none (*n* = 33), mild (*n* = 77), moderate (*n* = 83), and severe (*n* = 43) (Table 2). The cohort demonstrated significant age stratification across severity groups (75.4 ± 13.4 vs. 85.7 ± 6.6 years; *P* < 0.001), with 71.6% receiving PEG and 9.7% underwent TPN. PEG was the predominant method of percutaneous enteral nutrition in mild and moderate groups (75.3% vs. 69.9%) (Table 2). Median follow-up duration reached 314 days (IQR:114–635) for censored cases. Multivariable analyses revealed severity-dependent patterns cerebrovascular disease, severe dementia, aspiration pneumonia, PEG, and NT.CVC (*P* < 0.05). Mortality escalated progressively with worsening CONUT scores (18.2% vs. 79.1%, *p* < 0.001) (Table 2), aligning with caloric intake reductions (960.0 ± 150.3 vs. 846.3 ± 229.9 kcal/day, *P* = 0.011) (Table 2).

**Table 2:** Baseline characteristics of patients.

**Variables**	**Total (*n* = 236)**	**None (*n* = 33)**	**Mild (*n* = 77)**	**Moderate (*n* = 83)**	**Severe (*n* = 43)**	***P***
Age(year)	82.8 ± 9.3	75.4 ± 13.4	82.1 ± 8.8	85.1 ± 7.3	85.7 ± 6.6	< 0.001
Sex, *n* (%)						0.092
Male	95 (40.3)	8 (24.2)	28 (36.4)	40 (48.2)	19 (44.2)	
Female	141 (59.7)	25 (75.8)	49 (63.6)	43 (51.8)	24 (55.8)	
CI, *n* (%)	126 (53.4)	27 (81.8)	46 (59.7)	36 (43.4)	17 (39.5)	< 0.001
Dement, *n* (%)	97 (41.1)	5 (15.2)	26 (33.8)	43 (51.8)	23 (53.5)	< 0.001
Asp, *n* (%)	90 (38.1)	5 (15.2)	28 (36.4)	39 (47)	18 (41.9)	0.015
IHD, *n* (%)	42 (17.8)	4 (12.1)	11 (14.3)	15 (18.1)	12 (27.9)	0.223
PEG, *n* (%)	169 (71.6)	32 (97)	58 (75.3)	58 (69.9)	21 (48.8)	< 0.001
NT.CVC, *n* (%)	23 (9.7)	0 (0)	6 (7.8)	7 (8.4)	10 (23.3)	0.006
Oral, *n* (%)	14 (5.9)	4 (12.1)	4 (5.2)	4 (4.8)	2 (4.7)	0.497
Kcal.day	918.4 ± 190.4	960.0 ± 150.3	954.9 ± 178.9	905.4 ± 182.9	846.3 ± 229.9	0.011
Status, *n* (%)						< 0.001
Alive	105 (44.5)	27 (81.8)	37 (48.1)	32 (38.6)	9 (20.9)	
Death	131 (55.5)	6 (18.2)	40 (51.9)	51 (61.4)	34 (79.1)	
Conut	5.1 ± 3.1	0.5 ± 0.5	3.2 ± 0.8	6.3 ± 1.2	9.9 ± 1.0	< 0.001

CI, cerebrovascular diseases; Dement, severe dementia; asp, aspiration pneumonia; IHD, ischemic heart disease; NT.CVC, non-tunneled central venous catheters; PEG, percutaneous endoscopic gastrostomy; Oral, oral intake recovery; Conut, Controlling Nutritional Status.

### 3.2 Associations between CONUT and mortality

**Figure 1:**
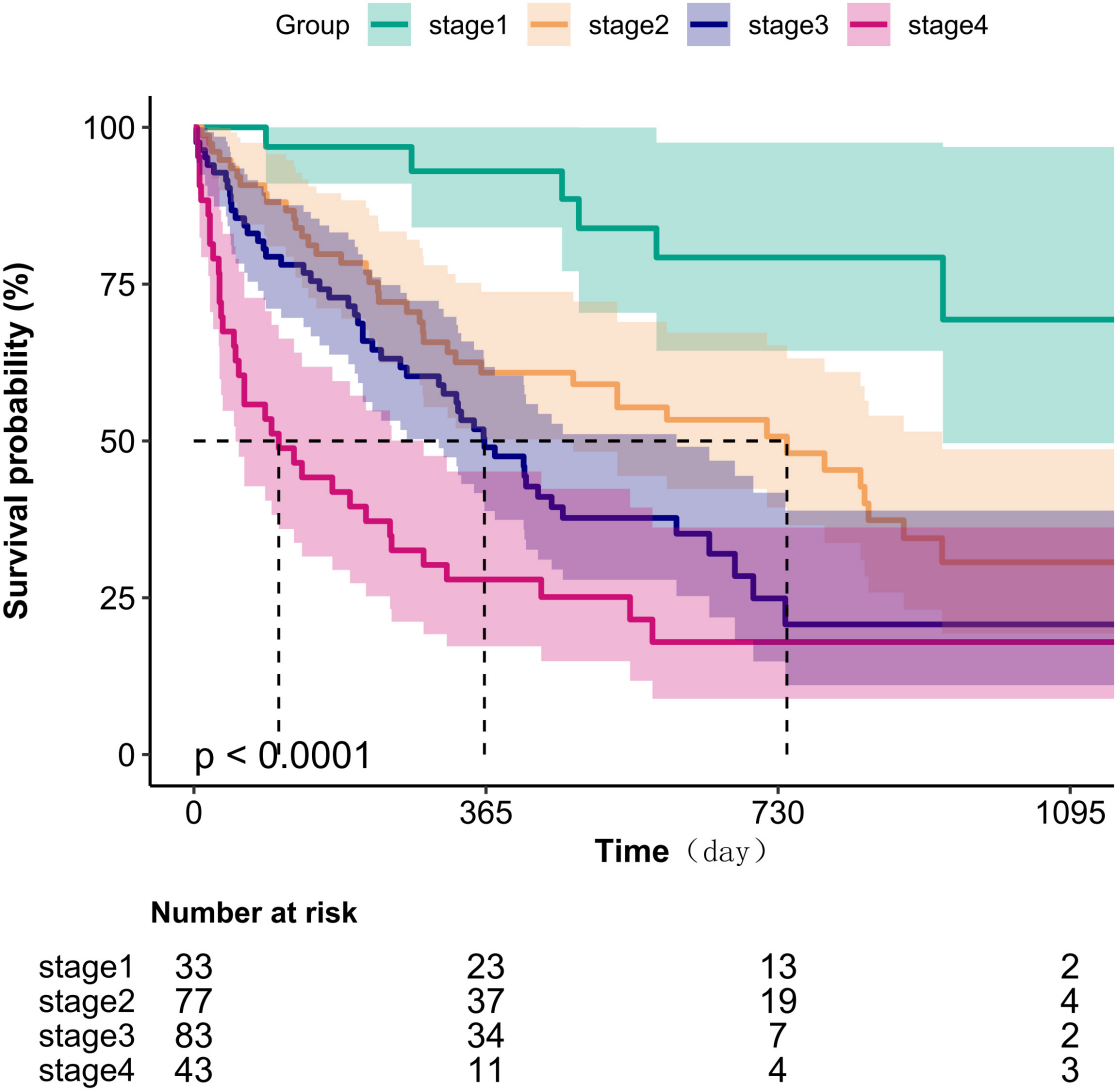
Kaplan–Meier survival analysis for mortality with CONUT in four groups.

**Figure 2:**
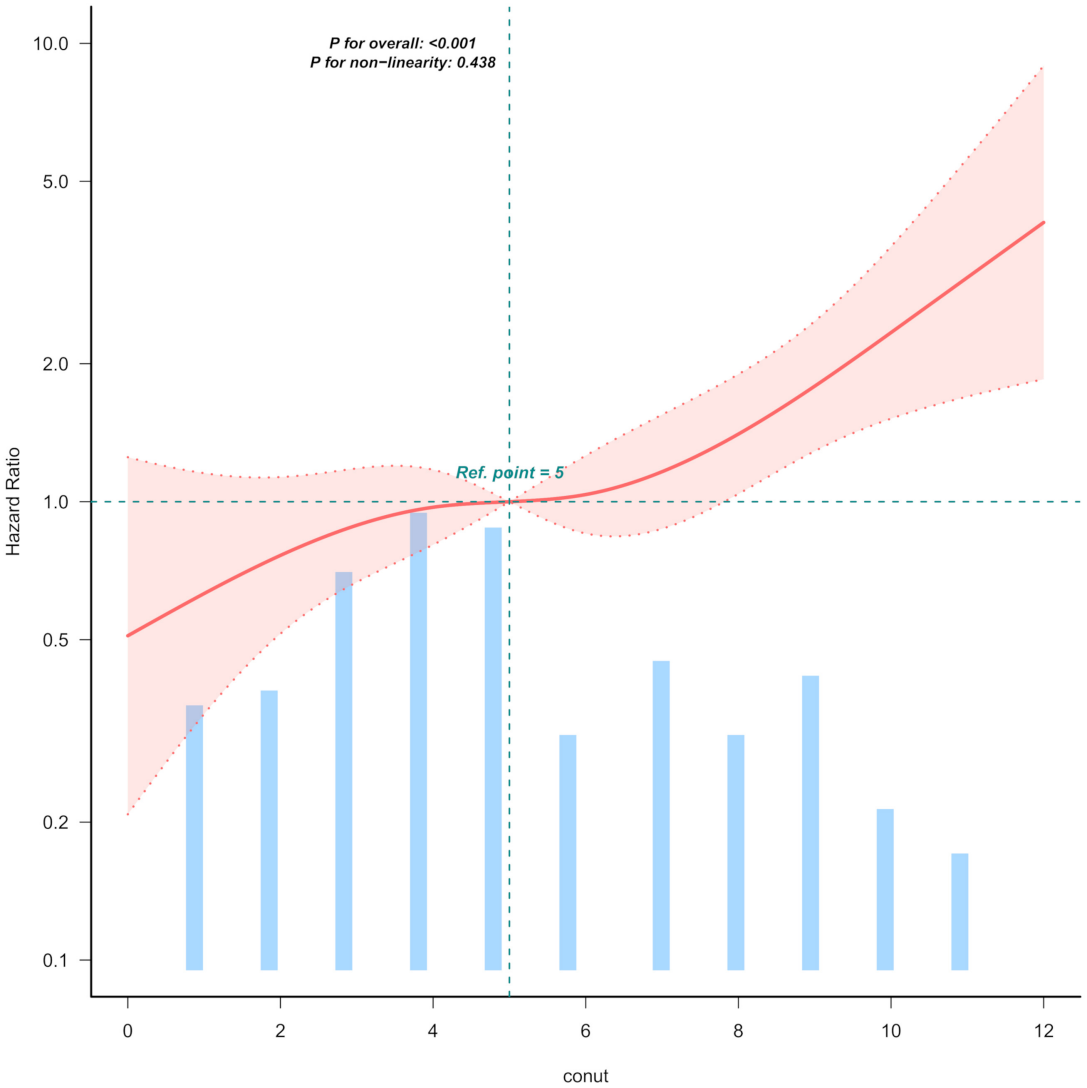
Linear dose-response relationship between CONUT and mortality of Japanese geriatric dysphagia patients. The figure presents multivariable adjusted hazard ratios (HRs) for mortality across varying levels of CONUT on a continuous scale. The solid deep red lines represent the multivariable-adjusted HRs. Light red areas depict the 95% confidence intervals derived from restricted cubic spline regressions with four knots. Dashed black lines serve as reference lines indicating no association at a hazard ratio of 1.0, recealing that mortality rises with increasing CONUT levels. The analysis includes 99.5% of the available data and adjusts for variables such as age, sex, ischemic heart disease (IHD), cerebrovascular diseases, severe dementia, aspiration pneumonia, percutaneous endoscopic gastrostomy (PEG), non-tunneled central venous catheters central venous catheter (NT.CVC), oral intake recovery and energy intake.

Kaplan-Meier survival analysis (Figure 1) demonstrated a gradient-dependent association between CONUT categories and mortality risk, with the severe malnutrition group (highest CONUT scores) exhibiting the most pronounced survival disadvantage (*P* < 0.0001). Complementing these findings, restricted cubic spline analysis (Figure 2) revealed a monotonic dose-response relationship between increasing CONUT scores and mortality risk in Japanese geriatric dysphagia patients (*P* for non-linear trend = 0.438).

Multivariable Cox regression analyses (Table 3) revealed a dose-dependent association between CONUT scores and mortality risk. When modeled as a continuous variable, CONUT demonstrated a robust positive association with mortality across all analytical frameworks (adjusted HR = 1.15 per 1-unit increment, 95% CI: 1.08–1.23; *P* < 0.001). Stratification by nutritional risk categories (Model 1) yielded incrementally increasing hazard ratios compared to the reference group (CONUT 0–1): mild (2–4) HR = 3.68 (95% CI: 1.56–8.68, *P* = 0.003), moderate (5–8) HR = 5.57 (95% CI: 2.38–13.05, *P* < 0.001), and severe malnutrition (9–12) HR = 9.53 (95% CI: 3.99–22.78, *P* < 0.001). Notably, this graded relationship persisted following comprehensive adjustment for covariates including demographic characteristics (age, sex), comorbidities (ischemic heart disease, cerebrovascular disorders, severe dementia), clinical complications (aspiration pneumonia), and nutritional parameters (percutaneous endoscopic gastrostomy [PEG] status, nasoenteric tube/catheter [NT.CVC] utilization, oral intake recovery, energy intake). Most importantly, dose-response relationship assessment via linear trend test confirmed monotonic risk escalation across CONUT categories (*P* < 0.001).

**Table 3:** Associations between CONUT and mortality in the multiple cox regression model.

**Variable**	**Model I**		**Model II**		**Model III**		**Model IV**	
	**HR (95% CI)**	***P*** **-value**	**HR (95% CI)**	***P*** **-value**	**HR (95% CI)**	***P*** **-value**	**HR (95% CI)**	***P*** **-value**
Conut	1.23 (1.16–1.3)	< 0.001	1.19 (1.12–1.27)	< 0.001	1.18 (1.11–1.25)	< 0.001	1.15 (1.08–1.23)	< 0.001
Conut.stage								
None (0–1)	1(Ref)		1(Ref)		1(Ref)		1(Ref)	
Mild (2–4)	3.68 (1.56–8.68)	0.003	2.67 (1.12–6.35)	0.026	2.73 (1.15–6.51)	0.023	2.46 (1.02–5.91)	0.045
Moderate (5–8)	5.57 (2.38–13.05)	< 0.001	3.1 (1.3–7.41)	0.011	2.98 (1.24–7.18)	0.015	2.91 (1.21–7.02)	0.017
Severe (9–12)	9.53 (3.99–22.78)	< 0.001	6.02 (2.48–14.6)	< 0.001	5.59 (2.3–13.6)	< 0.001	4.56 (1.84–11.3)	0.001
*P* for Trend		< 0.001		< 0.001		< 0.001		< 0.001

Model I: Unadjusted.

Model II: Adjusted for age, and sex.

Model III: Model II plus cerebrovascular diseases, severe dementia, aspiration pneumonia, ischemic heart disease.

Model IV:Model III plus non-tunneled central venous catheters, percutaneous endoscopic gastrostomy, oral intake recovery, energy intake.

**Figure 3:**
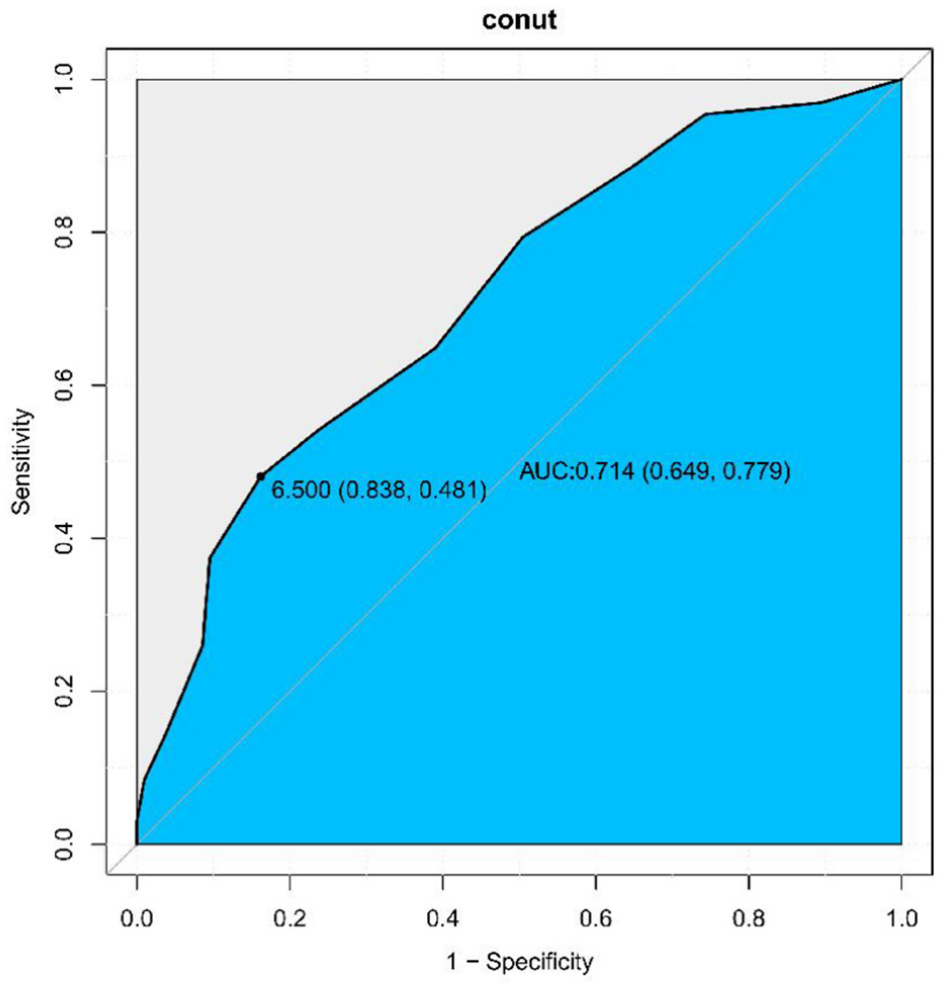
ROC-curve analysis of the CONUT score. The Area Under the Curve (AUC) was calculated as 0.714 (95% CI: 0.649–0.779). Sensitivity: 83.8%, Specificity: 48.1%.

**Figure 4:**
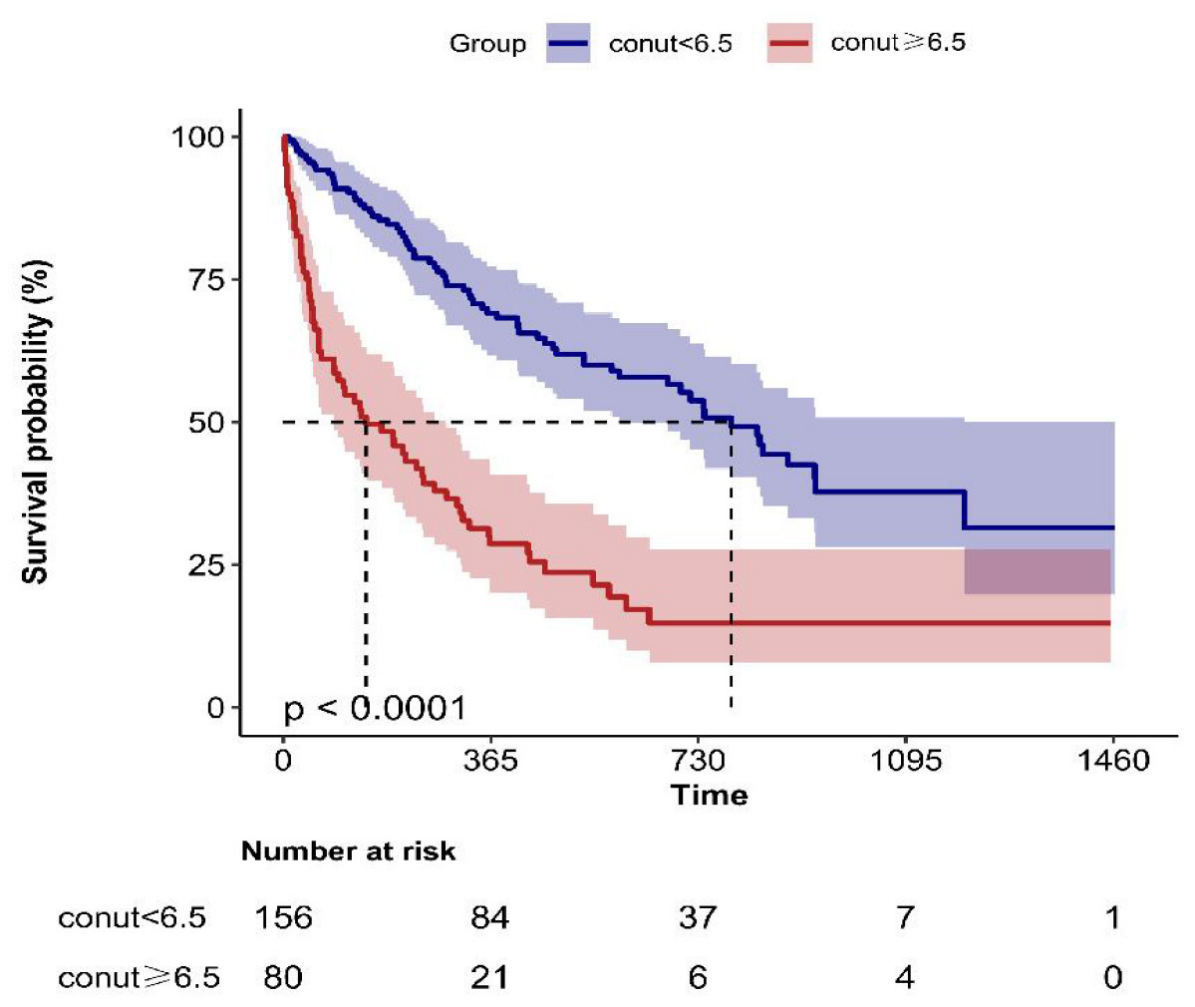
Kaplan–Meier survival curves for mortality.

Based on the above results, ROC analysis was employed to assess the predictive ability of biomarkers mortality in older patients with dysphagia. Our analysis indicated that the CONUT score achieved an AUC of 0.714 (95% CI: 0.649–0.779, Figure 3) for mortality prediction. Threshold analysis via the Youden index identified 6.5 as the optimal cut-off, with a sensitivity of 83.8% and specificity of 48.1% (Figure 3). Stratification by this value defined high-CONUT (≥6.5) and low-CONUT (< 6.5) cohorts. Kaplan-Meier curves (Figure 4) demonstrated substantially lower survival probability in the high- CONUT group vs. controls. Critically, multivariate Cox regression (Table 4) further established high nutritional risk as an independent mortality predictor (adjusted HR 2.12, 95% CI 1.48–3.04).

**Table 4:** Multivariate Cox regression analysis.

**Variable**	**Crude**		**Adjust**	
	**HR (95% CI)**	***P*** **-value**	**HR (95% CI)**	***P*** **-value**
Conut < 6.5	1(Ref)		1(Ref)	
Conut ≥ 6.5	3.08 (2.18–4.36)	< 0.001	2.12 (1.48–3.04)	< 0.001

Adjusted for age, sex,cerebrovascular diseases, severe dementia, aspiration pneumonia, ischemic heart disease,non-tunneled central venous catheters, percutaneous endoscopic gastrostomy, oral intake recovery, energy intake.

### 3.3 Subgroup analyses

**Figure 5:**
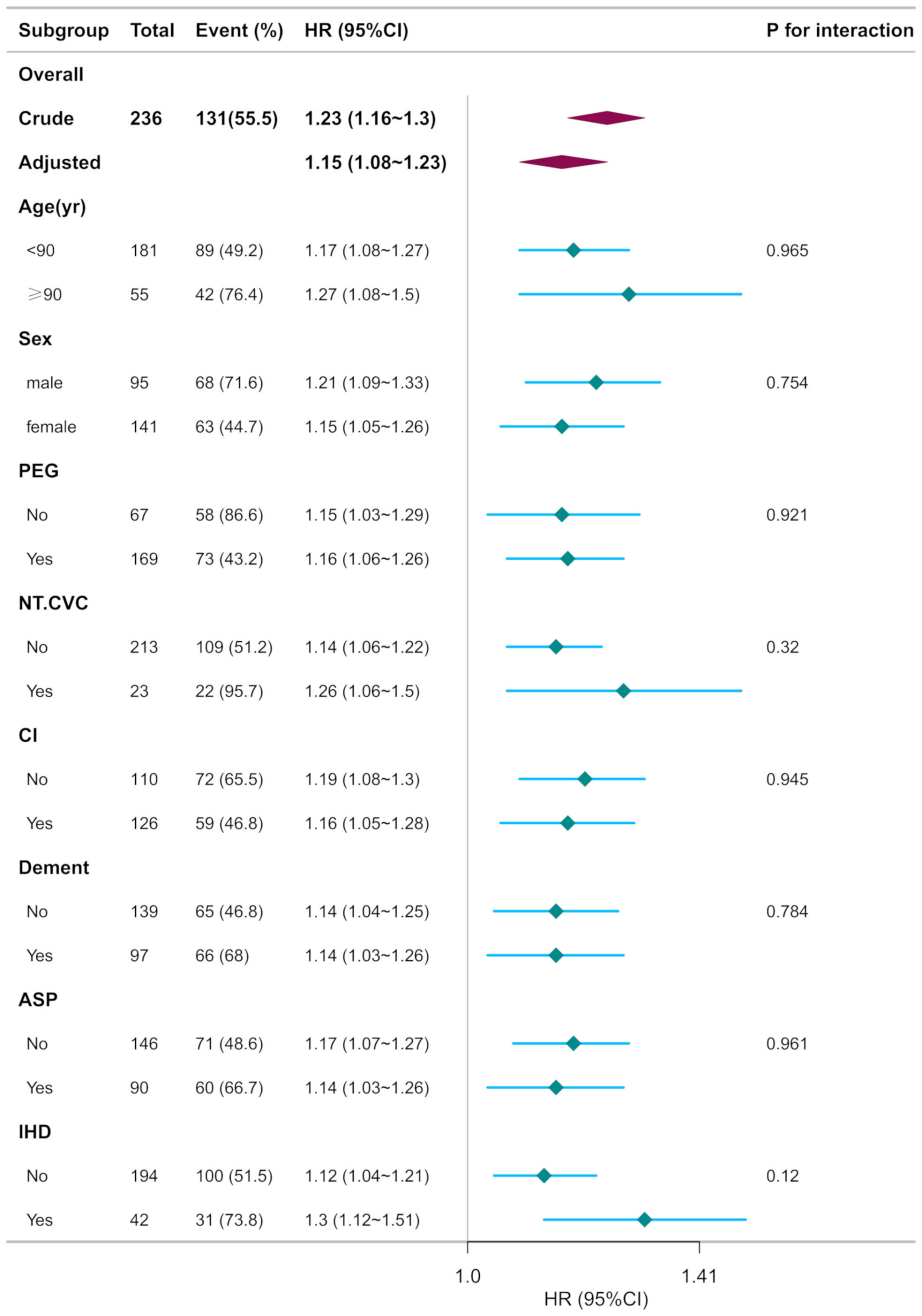
Subgroup analyses of CONUT associated with mortality. Hazard ratios (HRs) were adjusted for age, sex, cerebrovascular diseases, severe dementia, aspiration pneumonia, ischemic heart disease, non-tunneled central venous catheters, percutaneous endoscopic gastrostomy, oral intake recovery, energy intake.

Stratified analyses delineated in Figure 5 evaluated potential effect modifiers of the CONUT-mortality association. Predefined subgroups were categorized by: (a) demographic characteristics (age < 90 vs. ≥90 years, sex), (b) baseline comorbidities (cerebrovascular disorders, severe dementia, ischemic heart disease), (c) acute complications (aspiration pneumonia), and (d) clinical interventions (non-tunneled central venous catheter [NT.CVC] placement, percutaneous endoscopic gastrostomy [PEG]). Consistent effect estimates were observed across all strata (age, *P* = 0.965; sex, *P* = 0.754; PEG, *P* = 0.921; NT.CVC, *P* = 0.32; cerebrovascular disorders, *P* = 0.945; severe dementia, *P* = 0.784; aspiration pneumonia, *P* = 0.961; ischemic heart disease, *P* = 0.12; all interaction *p*-values >0.05), suggeting the prognostic robustness of CONUT scores across heterogeneous clinical profiles (Figure 5).

## 4 Discussion

In our investigation, we identified a gradient-dependent correlation between the CONUT score and mortality risk in a cohort of elderly individuals with dysphagia in Japan. Specifically, we observed that each incremental rise in the CONUT score was linked to a 15% increase in the overall risk of mortality (HR 1.15, 95% CI 1.08–1.23). Notably, individuals with a CONUT score ranging from 9 to 12 points (indicative of severe malnutrition) faced a mortality risk 9.5 times higher than those without malnutrition (HR 9.53, 95% CI 3.99–22.78). Even after accounting for all covariates, the severe malnutrition group exhibited a mortality rate 4.56 times higher than that of the adequately nourished group (*P* = 0.001). Importantly, this dose-response relationship persisted following adjustments for potential confounders such as age, gender, stroke, dementia, aspiration pneumonia, and feeding method. These findings underscore the utility of CONUT as an objective prognostic tool for early assessment in this demographic.

Dysphagia, a common geriatric syndrome, significantly affects independence, quality of life, and mortality rates among the elderly (4, 22). Its true incidence is often underestimated due to subtle symptoms and silent aspiration, with dysphagia being a primary pathophysiologic factor leading to aspiration pneumonia in this population (2). Research in indicates that 10.3% of acute ischemic stroke patients with dysphagia develop stroke-associated pneumonia (23). Elderly individuals with dysphagia may experience reduced food intake, potentially leading to dehydration and malnutrition (24).The prevalence of malnutrition rises from 58.9% to 78.9% in acute and subacute stroke patients with dysphagia, respectively (25). Moreover, our findings suggest that an increase in the CONUT score, correlates with a decrease in the actual energy intake from 960 kcal/day to 846 kcal/day (*P* = 0.011), highlighting the energy gap as a critical factor in the rapid progression of malnutrition.

CONUT is more suitable for individuals with dysphagia compared to conventional assessment tools. Unlike questionnaires such as Mini Nutritional Assessment Short-Form(MNA-SF)that necessitate patient cooperation, CONUT relies solely on three blood indicators (albumin, lymphocytes, and cholesterol), making it feasible for patients with cognitive impairments or those unable to orally consume food (11, 26). CONUT provides an objective alternative that does not require patient cooperation. The Geriatric Nutritional Risk Index (GNRI) is a practical tool commonly used in clinical settings, to evaluate the nutritional risk in elderly patients. It involves assessing body weight, height, and serum albumin levels. GNRI < 91.2 was found to be independently and significantly correlated with a higher risk of dysphagia (OR 3.094; CI 1.057-9.058; *P* = 0.039) (27). In contrast to tools like GNRI and Subjective Global Assessment (SGA) that rely on subjective parameters such as weight fluctuations or dietary recall, CONUT is solely based on objective laboratory parameters, reducing potential biases. Consequently, CONUT is well-suited for rapid and dynamic nutritional status assessments (27–29).

A gap exists in current research literature concerning the utilization of CONUT for evaluating the prognosis of elderly individuals with dysphagia.This study categorized patients based on CONUT scores to examine variations between individuals with adequate nutritional status and those experiencing moderate to severe malnutrition. Our findings indicated as a gradual increase in the risk of mortality with higher CONUT scores (*P* < 0.001). Patients with a CONUT score of 9–12 points (indicative of severe malnutrition) exhibited a significantly higher risk of death(79.1%,*P* < 0.001). These results align with prior studies involving cardiovascular, oncological, and general elderly inpatients (10, 12–14, 30). Notably, our study specifically focused on a subset of patients with dysphagia, a group at elevated risk of malnutrition, suggesting that dysphagia may exacerbate adverse consequences of malnutrition. This observation underscores the critical influence of nutritional status on the prognosis of elderly patients with dysphagia. Malnutrition is not only a result of disease but can also exacerbate disease progression, compromising immune function, muscle loss, and overall physiological deterioration (31–33). Elderly individuals with dysphagia encounter difficulties in maintaining adequate nutritional intake, and malnutrition can further compromise immune response, escalating susceptibility to infections, complications, and mortality (3, 5, 6). Hence, timely detection and management of malnutrition play a pivotal role in enhancing the clinical outcomes of these patients.

Prior research has demonstrated an inverse correlationg between Prognostic Nutritional Index (PNI),derived from the albumin and total lymphocyte count, and mortality in elder Japanese patients with dysphagia (HR = 0.94, 95% CI: 0.92–0.97, *P* < 0.001) (34). Multivariable Cox regression analyses in our investigation established CONUT as an autonomous mortality predictor among elderly dysphagia patients. Notably, escalating CONUT values correlated with progressively heightened mortality risk. ROC curve evaluation further confirmed that CONUT maintained discriminative equivalence to the PNI in predicting all-cause mortality (ROC curve AUC = 0.714 vs. 0.721, *P* = 0.665, Figure 3 and Supplementary Figure 1).

Compared with COUNT, PNI lacks the inclusion of total cholesterol as a parameter. Machine learning analysis has highlighted total cholesterol as the most critical factor for assessing nutritional status (35). Research suggests that cholesterol plays a role in inflammatory responses to infections and chronic metabolic inflammatory diseases like atherosclerosis and obesity (36). Moreover, cholesterol metabolism has been shown to enhance the anti-tumor response of CD8(+) T cells (37). Therefore,CONUT may detect lipid metabolism disorders overlooked by PNI, making it a more sensitive tool for identifying long-term malnutrition in elderly dysphagia patients.

In patients with mild or moderate malnutrition, percutaneous endoscopic gastrostomy (PEG) was the predominant method of percutaneous enteral nutrition (75.3%; 69.9%), aligning with prior research. Variations in nutritional administration routes (enteral vs. parenteral) may influence outcomes (17). Subgroup analyses were independently performed for PEG and NT.CVC patients, yielding consistent effect estimates (*P* = 0.921; *P* = 0.32). Among patients with dysphagia, irrespective of the type of nutritional supplementation (enteral or parenteral), CONUT emerged as an independent mortality risk factor. Subgroup analyses, stratified by age, gender, ischemic heart disease (IHD), cerebrovascular disorders, severe dementia, and aspiration pneumonia, showed consistent findings without significant interactions. This suggests that monitoring COUNT and delivering tailored nutritional interventions based on individual nutritional status may improve survival outcomes, even in patients with multiple comorbidities. Subsequent studies should place greater emphasis on the prognostic utility of the CONUT score at various time intervals and explore the effects of nutritional interventions on the prognosis.

CONUT is a rapid and straightforward screening tool utilized for assessing nutritional status rely on common blood test indicators like ALB, total cholesterol, and total lymphocyte count in clinical settings. Our research suggests that these tests, being cost-effective and efficient, could serve as crucial prognostic markers, advocating for their broader integration into clinical practice.

## 5 Limitations

However, this study is constrained by its single-center design and limited sample size, potentially introducing selection bias. Furthermore, it overlooks various factors like socioeconomic status and lifestyle that could impact patient prognosis, indicating the need for further comprehensive investigations. Finally, the timeframe (2014–2017) is relatively old and may not fully reflect current clinical nutrition practices. Future studies we will increase the sample size, adopt a multi-center research design, and include more potential confounding factors to further verify the predictive value of the CONUT score.

## 6 Conclusion

The CONUT score demonstrated a gradient-dependent association with mortality among Japanese geriatric patients with dysphagia. Subgroup analyses revealed no significant interaction effects. These findings indicate that the CONUT score may function as a clinically useful tool for risk stratification in dysphagia patients, facilitating early detection of high-risk individuals and informing personalized clinical interventions.

## Data Availability

The datasets presented in this study can be found in online repositories. The names of the repository/repositories and accession number(s) can be found in the article/Supplementary material.
