# Supplementary figures and images for: Association between controlling nutritional status score and mortality in older patients with dysphagia in Japan: a retrospective cohort study
Source: Front Nutr. 2025 Nov 18;12:1682772. doi: 10.3389/fnut.2025.1682772 (PMC12668998; doi:10.3389/fnut.2025.1682772)

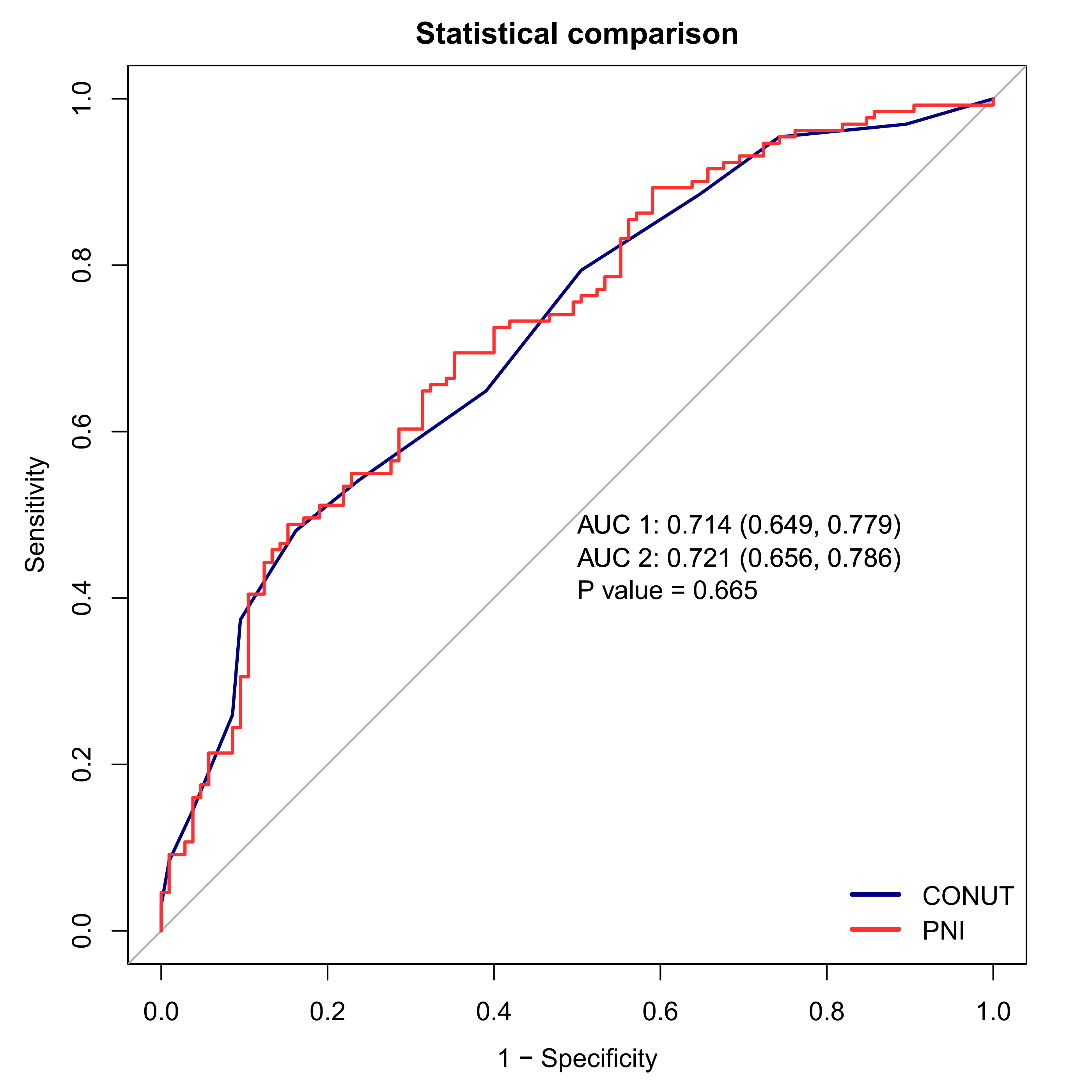

Supplement: Supplementary file 2 [file Image_1.tif]
